# Avoiding monetary loss: A human habenula functional MRI ultra-high field study

**DOI:** 10.1016/j.cortex.2021.05.013

**Published:** 2021-09

**Authors:** Kathrin Weidacker, Seung-Goo Kim, Camilla L. Nord, Catarina Rua, Christopher T. Rodgers, Valerie Voon

**Affiliations:** a Department of Psychiatry, University of Cambridge, Addenbrooke's Hospital, Cambridge, United Kingdom; b Department of Psychology, Swansea University, Singleton Campus, Swansea, United Kingdom; c Department of Psychology and Neuroscience, Duke University, Durham, NC, United States; d MRC Cognition and Brain Sciences Unit, University of Cambridge, Cambridge, United Kingdom; e Wolfson Brain Imaging Centre, University of Cambridge, Cambridge Biomedical Campus, Cambridge, United Kingdom

**Keywords:** Habenula, Reward processing, Loss avoidance, Monetary incentive delay task, Functional connectivity

## Abstract

A number of convergent human neuroimaging and animal studies suggest that habenula neurons fire in anticipation of non-rewarding outcomes, and suppress their firing in anticipation of rewarding outcomes. This normative function of the habenula appears disrupted in depression, and may be critical to the anti-depressant effects of ketamine. However, studying habenula functionality in humans using standard 3 T MRI is inherently limited by its small size. We employed ultra-high field (7 T) fMRI to investigate habenular activity in eighteen healthy volunteers during a Monetary Incentive Delay Task, focussing on loss avoidance, monetary loss and neutral events. We assessed neural activation in the field of view (FOV) in addition to ROI-based habenula-specific activity and generalized task-dependent functional connectivity. Whole FOV results indicated substantial neural differences between monetary loss and neutral outcomes, as well as between loss avoidance and neutral outcomes. Habenula-specific analyses showed bilateral deactivation during loss avoidance, compared to other outcomes. This first investigation into the habenula's role during loss avoidance revealed that the left habenula further differentiated between loss avoidance and monetary loss. Functional connectivity between the right habenula and the ipsilateral hippocampus and subcallosal cingulate (regions implicated in memory and depression pathophysiology) was enhanced when anticipating potential losses compared to anticipating neutral outcomes. Our findings suggest that the human habenula responds most strongly to outcomes of loss avoidance when compared to neutral and monetary losses, suggesting a role for the habenula in both reward and aversive processing. This has critical relevance to understanding the pathophysiology of habenula function in mood and other neuropsychiatric disorders, as well as the mechanism of action of habenula-targeting antidepressants such as ketamine.

## 1 Introduction

The human habenula (HB) complex plays a critical role in anti-reward processing and is relevant also to reward processing (Matsumoto and Hikosaka, 2007, 2009). The lateral habenula (LHB) has been implicated in the pathophysiology of psychiatric disorders such as Major Depressive Disorder (MDD) (Lawson et al., 2017; Sartorius et al., 2010; Yang et al., 2018a; Zhang et al., 2019), with rodent studies highlighting the LHB in mediating the anti-depressive effect of ketamine (Yang et al., 2018b). The human HB is located bilaterally between the pineal gland and the dorsomedial thalamus and represents a small neural structure (around 30 mm^3^ volume per hemisphere) (Lawson et al., 2013) composed of grey and white matter. Cytologically, the HB can be subdivided into medial and lateral nuclei (Akagi & Powell, 1968) which have different genetic profiles as well as anatomic connections based on non-human studies (Namboodiri et al., 2016). The possibility to differentiate medial and lateral HB in human *in vivo* neuroimaging research is limited due to their small size (Epstein et al., 2018). Here we aim to assess HB function using a task-based ultra-high field 7 T fMRI study in healthy volunteers focusing on loss avoidance and incurring losses.

Non-human primate research suggests the LHB plays a critical role in the upstream modulation of midbrain dopaminergic neurones and is involved in anti-reward processing and, to a lesser extent, in reward processing (Matsumoto and Hikosaka, 2007, 2009). When non-human primates are anticipating non-rewarding outcomes, excitation of the LHB neurons temporally precedes the inhibition of dopamine neurons. Similarly, electric stimulation of LHB neurons induced inhibition of dopamine neurons, indicating that the inhibitory effect of non-rewarding stimuli on dopamine neurons is guided by the LHB. The anticipation of rewarding outcomes, on the other hand, leads to decreased firing of LHB neurons (Matsumoto & Hikosaka, 2007). A similar neuronal response profile is seen in response to the outcomes themselves, with strong excitation of LHB neurons for negative and inhibition for positive outcomes (Matsumoto & Hikosaka, 2009). Single-cell recordings further showed that the neuronal response of the LHB depends on the context, with strongest neuronal responses to cues predicting the worst outcome among the available alternatives (for instance: the absence of reward when the alternative is reward, or the presence of punishment when the alternative is absence of punishment) (Matsumoto & Hikosaka, 2009). The LHB neurons are also sensitive to the mismatch between prediction and outcome as indicated by weaker excitatory responses when a negative outcome was fully predictable compared to a less certain outcome, and, greater negative prediction errors being associated with increased LHB neuron firing rate (Matsumoto & Hikosaka, 2009). Thus, the LHB is responsive to both reward and negative prediction errors, and in particularly to negative motivational value, with the encoding direction opposite to that of dopamine neurons (Matsumoto and Hikosaka, 2007, 2009).

In humans, neuroimaging research using task-based functional magnetic resonance imaging (fMRI) to delineate the HB function is sparse due to the inherent difficulty of isolating signal change from small structures such as the habenula. Similarly, the majority of fMRI studies on the human HB were carried out using standard field strengths, such as 3 T, which further limits the delineation of the HB due to lower resolution than that afforded by ultra-high field imaging (7 T). Indeed, the standard voxel size used in 3 T EPI sequences for fMRI studies is 3 mm, a major limitation for a nucleus of 30 mm^3^ volume. Despite these constraints, previous human fMRI studies (including those using specialised high-resolution 3 T sequences) converge with primate single-cell recordings demonstrating the importance of the human HB for the anticipation of punishment and changing reward contingencies.

In healthy humans, anticipating electrical shocks (*vs* neutral outcomes) evoked increased activation in bilateral insula, caudate, but also in the left, and to a lesser extent, in the right, HB (Hennigan et al., 2015). Region of interest (ROI) analyses revealed that the left-hemispheric HB activity increase during punishment anticipation also holds when compared to reward (juice receipt) anticipation; anticipating rewarding versus neutral outcomes did not affect left HB signal change (Hennigan et al., 2015). In line with primate findings, the human HB is also sensitive to probabilities. For example, cues predicting a high versus low chance of losing points in a guessing game evoked increased left-, but not right-, hemispheric HB activation (Furman & Gotlib, 2016). While this previous study found the left HB to be sensitive to probabilities, a separate study investigating HB responses to cues indicative of a high versus low chance for upcoming punishment (in the form of electrical shocks) or monetary rewards reported a bilateral increase in HB activation for the anticipation of punishment and a decrease for monetary rewards. Interestingly, this study also investigated monetary losses within the same design and while HB activation increased, the HB response to losses fell in between monetary wins and shock as punishment, in line with earlier reports of the HB responding to the most salient among outcomes (Lawson et al., 2014; Matsumoto & Hikosaka, 2009).

Converging with the HB's role in tracking prediction errors in non-human primates, human *in vivo* neuroimaging research suggests that right HB activation increases linearly in response to increased adversity of anticipatory cues, highlighting the sensitivity of the human HB to the motivational value of anticipatory cues (Lawson et al., 2014). When utilizing losing and winning points as punishment and reward conditions*,* no laterality effect was observed regarding prediction errors, instead, bilateral HB activation was enhanced for punishment-related prediction errors compared to reward-based prediction errors (Liu et al., 2017). Investigating neural responses to prediction errors across studies, a recent Activation Likelihood Estimation (ALE) meta-analysis confirmed a role for the human HB particularly for punishment-related prediction errors, in addition to brain areas such as the middle frontal gyrus (MFG) and the insula. Reward-based prediction errors on the other hand, were associated with activation changes in reward-processing related brain areas such as the striatum including the nucleus accumbens (NACC) (Garrison et al., 2013).

While the reviewed findings relate to the anticipation of positive and negative outcomes, differential HB activation has also been observed during outcome presentation. When assessing HB responses to outcomes in a guessing game, bilateral HB responses increased for monetary losses over wins, while only left-hemispheric signal change differed between healthy volunteers and patients suffering from MDD (Furman & Gotlib, 2016). In contrast, when investigating HB response to outcomes such as loosing or winning points (each normalized to neutral outcomes), left-hemispheric HB activation differentiated between punishment, which led to increased, and reward, which resulted in decreased HB activation (Liu et al., 2017). Regarding the presentation of negative and positive non-verbal feedback during a prediction task, enhanced bilateral thalamus activation, which included the HB, was reported for negative versus positive feedback alongside greater activity in bilateral anterior insula and anterior cingulate cortex (ACC) (Flannery et al., 2019).

In summary, convergent studies of human HB function using 3 T highlight HB responsiveness to punishment and reward, although laterality effects remain unclear. During anticipation, as well as during outcome presentation, the human HB increases activation for stimuli associated with punishment over reward. This is convergent with non-human primate research investigating the firing patterns of LHB neuron populations, which showed that HB subpopulations are activated following negative and inhibited following positive motivational stimuli (Matsumoto & Hikosaka, 2009).

While task designs differed across studies, most investigations utilized some form of delayed anti-/reward paradigm, in which negative outcomes are related to losing points, money or punishment. However, whether the human HB also responds differentially to loss avoidance, the absence of incurring losses, has not yet been investigated. Neurally, the anticipation of loss avoidance differs from that of reward anticipation, with anticipation of loss avoidance evoking less signal change in the NACC in children (Bjork et al., 2008). Similarly, while ventral striatal activation was generally decreased for the anticipation of loss avoidance compared to rewards in healthy adolescents, adolescents with Attention-deficit/Hyperactivity disorder (ADHD) expressed reduced ventral striatal activation during reward anticipation, but not during loss avoidance anticipation when compared to controls, further highlighting the differences underlying reward and loss avoidance during anticipation (Scheres et al., 2007). Additional differences between loss avoidance and reward were reported during the outcome phase. In adults, neural responses to feedback relating to the successful avoidance of losses versus neutral outcomes increased activation in the inferior frontal gyrus and the cerebellum while clusters being more activated to rewarding than neutral outcomes were more widespread, including caudate, globus pallidus, and cingulate brain regions among others (Filbey et al., 2013). Similarly, neural activity during loss avoidance, but not reward outcomes, differentiated between controls and adults unmedicated for childhood ADHD, with reduced bilateral insular and precentral gyrus activity in the latter group for loss avoidance outcomes (Stoy et al., 2011).

Previous research comparing successful loss avoidance to loss incurrence, instead of reward, did not focus on HB activation, but uncovered enhanced activation for loss avoidance in superior temporal gyri, pre/-cuneus, and reward-related subcortical brain regions such as the caudate and the ventral striatum in healthy adults (Beck et al., 2009). The concept of loss avoidance is further of relevance for disorders of addiction. Comparing loss avoidance to loss incurrence between alcohol-dependent patients and controls revealed enhanced activation in reward-related areas such as ventral striatum, caudate, and putamen as well as in insula, temporal gyri, MFG and precuneus in controls, while neural activation during reward trials did not differ between groups (Beck et al., 2009). A separate investigation confirmed the reduced striatal activation in alcohol-disordered patients during successful versus non-successful loss avoidance, and additionally revealed aberrant loss avoidance processing in pathological gamblers, with reduced activation in ventral striatum and medial prefrontal cortex compared to controls (Romanczuk-Seiferth et al., 2015).

Loss avoidance is of high relevance to mental disorders such as ADHD, but also plays and important role in addiction, particularly for alcohol abuse and gambling disorders. Neurally, loss avoidance differs from reward processing during anticipation and receipt, and to incurring losses during the feedback phase. Experimentally, most reviewed research utilized versions of the Monetary Incentive Delay (MID) paradigm (Knutson et al., 2001). During each trial, a cue is presented which indicates the upcoming trial type, e.g., punishment or reward. This is followed by a variable delay during which anticipation of the trial outcome occurs. Thereafter participants are asked to correctly perform a task, for instance, to press a button corresponding to the direction of an arrow. This response phase is typically dynamically adjusted to enhance task difficulty and to increase the proportion of errors [e.g., (Mei et al., 2018; Miller et al., 2014; Nestor et al., 2017; Zhang et al., 2017)]. Thereafter, visual feedback relating to the correctness of their response is presented, termed outcome phase. The MID is especially suited for studying the neural basis of anticipation and receipt due to separating the anticipation and outcome phases in time.

Given the importance of loss avoidance for psychiatric disorders, we here report the first results on human HB function in relation to loss avoidance in healthy volunteers, making use of advanced neuroimaging techniques, ultra-high field imaging (7 T). We utilize the MID task with Loss and Neutral trials, separating monetary loss avoidance and loss incurrence during the outcome phase. We additionally investigate generalized task-dependent functional connectivity involving the HB.

## 2 Methods

### 2.1 Participants

Twenty right-handed participants (10 male) participated in the study. All participants fulfilled 7 T scanning safety criteria and reported no previous brain injuries, seizures, and mental health diagnoses. All participants provided informed consent before participation and the study was approved by the local ethics committee. Two females were excluded, one due to the HB moving out of the field of view (FOV), the second due to technical issues. The resultant sample of eighteen participants had a mean age of 29.78 years, age ranged between 20 and 42 years. Sample size was determined based on previous 7 T studies and inclusion/exclusion criteria were established prior to data analyses.

### 2.2 Monetary incentive delay (MID) task

**Fig. 1:**
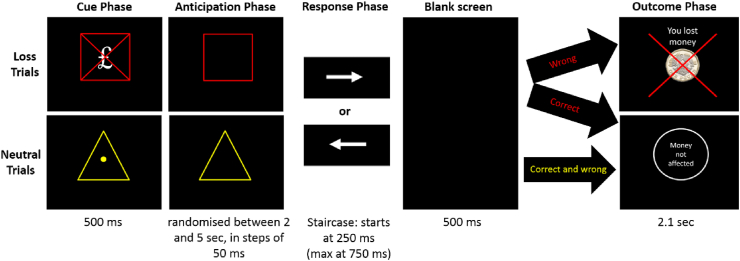
Durations and stimuli used during the different Monetary Incentive Delay Task phases per Loss and Neutral trials. The response phase was adjusted using the staircase procedure, independently per trial type. Correct responses decreased the response phase duration of the next trial of the same trial type by 50 msec, incorrect responses increased that response phase duration by 50 msec. In the Loss trials, incorrect/late responses were followed by “You lost money” during the outcome phase, while correct responses within the allowed response window led to a neutral outcome screen, “Money not affected”. During Neutral trials, the latter outcome screen was shown regardless of response correctness.

The MID task was programmed using Presentation software (version 20.2, www.neurobs.com) and responses were recorded using an fMRI-compatible button box. The MID task consisted of two trial types: 40 Loss and 30 Neutral trials, shown in equal proportions in the first and second half of the task. Trial presentation was pseudo-randomised with the restriction of less than three subsequent trial type repetitions. Each trial consisted of fives phases: cue, anticipation, response, blank screen, and outcome phase (see Fig. 1 for stimuli and durations). Participants were instructed that incorrectly responding during Loss trials results in losing money, and that if they do not lose much money during this task, they will receive an additional £5 at the end of the experiment, in addition to their payment for participation which was £10 per hour. For Neutral trials, participants were instructed that correctness of response does not affect their pay. In reality, all participants received the additional £5 at the end of the experiment. In the first part of the trial, the Cue phase, participants were shown a visual cue (red square with a crossed out £ sign for Loss trials; Yellow triangle with a dot in the centre for Neutral trials) indicating the upcoming trial type. During the anticipation phase, a slightly altered version of the Cue was shown (empty red square for Loss trials; Empty yellow triangle for Neutral trials). During the response phase, an arrow was either pointing to the left (requiring a button press with the right index finger) or to the right (requiring a button press with the right middle finger). Left and right arrows were presented equally often per trial type, pseudo-randomised with the restriction of less than four trials requiring the same response in a row. All responses were required to be as fast and accurate as possible. To enable analyses of correct (pressing the correct button within the response time window) and wrong responses (pressing the wrong button within the response time window, misses, or any responses outside the response window), the duration of the response window was constantly adjusted to yield 50% correct responses, in line with previous MID task designs (Mei et al., 2018; Miller et al., 2014; Nestor et al., 2017; Zhang et al., 2017). The initial response window was set to 250 msec and adjusted using an independent staircase procedure per trial type. Incorrect responses prolonged the response window by 50 msec, correct responses decreased the allowed response duration by 50 msec. Due to the staircase procedure, button presses occurring outside the response window were likely. To avoid contamination of the outcome phase by button presses, a blank screen (500 msec duration) interspersed the response phase and the outcome phase. What was shown during the outcome phase of Loss trials depended on the correctness of the response during the response phase: Correct button presses led to showing “Money not affected”, indicating successful loss avoidance, and wrong/late responses led to a screen with a crossed-out pound coin accompanied by “You lost money”, indicating monetary loss. The outcome phase for Neutral trials was independent of the correctness of responses and led for both correct and wrong/late button presses to the “Money not affected” screen.

Between trials, a fixation cross was shown. The duration of the fixation cross, as well as the duration of the anticipation phase, were each drawn randomly from two independent (one for fixations and one for anticipation) discrete uniform distributions (each from 2000 to 5000 msec, in steps of 50 msec) without replacement, except for 9 additional symmetrical samplings (the mean: 3500 msec, the four shortest: 2000, 2050, 2100, 2150 msec, and four longest durations: 4850, 4900, 4950, 5000 msec) to conform with the number of trials.

### 2.3 Image acquisition

Scanning was performed using the 7 T Terra MRI scanner (Siemens, Erlangen, Germany) at the Wolfson Brain Imaging Centre, Cambridge, UK and a 32-channel receive (1Tx/32Rx) head coil (Nova Medical Inc, MA, USA).

To obtain a high-quality uniform T1w image, the Magnetization Prepared with 2 Rapid Gradient Echoes (MP2RAGE) sequence was used (Marques et al., 2010) with the following parameters: TR/TE = 4300/1.99 msec, TI1/TI2 = 840/2370 msec, nominal FA1/FA2 = 5/6°, in-plane resolution = .75 × .75 mm^2^, .75 mm slice thickness, image matrix = 300 × 320, 224 slices, GRAPPA acceleration factor = 3, bandwidth = 250 Hz/pixel.

**Fig. 2:**
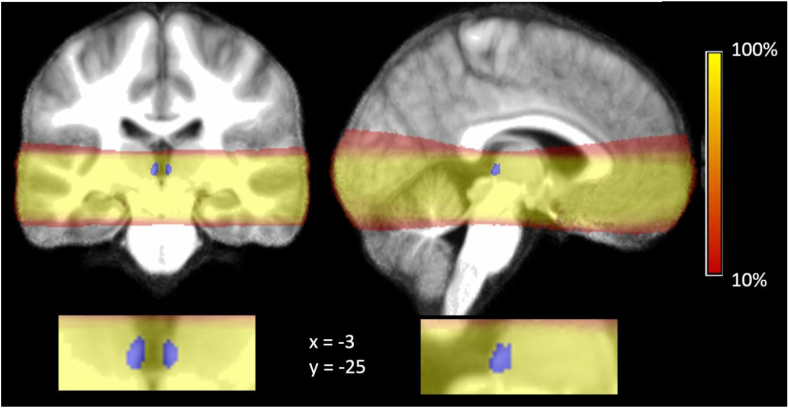
FOV overlap across participants. Shown is the percentage overlap across all participants (from 10 to 100%) for the acquired FOV in MNI space (x = −3, y = −25). The habenula is shown in blue in the FOV as well as in an enlarged cut-out of the habenula location.

The functional data was acquired using a .8 mm isotropic 2D single-band gradient-echo echo-planar imaging (GE-EPI) sequence: TR/TE = 3000/22 msec; nominal FA = 77°, 36 slices, no slice gap, image matrix = 256 × 256, GRAPPA acceleration factor = 3, bandwidth = 1028 Hz/pixel, phase-encoding direction anterior-posterior (A-P), partial Fourier = 5/8. Five volumes were collected with the same parameters as the functional scan but with phase encoding reversed (P-A) before task onset for B_0_ distortion correction. During scanning, the MP2RAGE T1 maps were visualised for each individual to localise the HB. The FOV was angled for each subject to ensure the HB was located at least 5 slices above the lower border of the FOV and that the FOV extended as far as possible into the ventromedial prefrontal cortex (see Fig. 2), as such the FOV tilt angles varied minimally across participants.

To correct for physiological fluctuations in the fMRI data, cardiac and respiratory data were recorded from the scanner's pulse oximeter on the left index finger and a pneumatic belt around the diaphragm.

### 2.4 Image pre-processing

SPM12 (Wellcome Trust Centre for Neuroimaging, London, UK; http://www.fil.ion.ucl.ac.uk/spm) was utilized for all preprocessing steps unless specified otherwise. Structural images were skull-stripped and bias-corrected using SPM12's unified segmentation approach (Ashburner & Friston, 2005) and normalised to MNI space. For creation of the study-specific 7 T template, all normalised T1w images were averaged using SPM12's ‘imcalc’.

For the fMRI data, field maps were created using FSL's ‘topup’ routine (http://fsl.fmrib.ox.ac.uk/fsl/fslwiki/TOPUP) to reduce geometric distortions based on five images of each phase encoding direction. The functional data were realigned and unwarped, slice timing corrected, the mean images were bias-corrected using the unified segmentation approach (Ashburner & Friston, 2005) and coregistered to the individual's T1w in native space.

For the HB ROI analyses, separate left and right HB masks were created by manually selecting each individual habenular voxel on each individual's T1w volume (in native space) using the software MRIcron (Rorden & Brett, 2000). Given the high contrast of the 7 T MP2RAGE, HB delineation was based on visual inspection of T1w image intensity. We additionally created HB ROI's based on the geometric method (Lawson et al., 2013), outlined in the supplement and Supplementary Figure 1, as additional support for our results. A visual example of the derived masks based on image intensity and the geometric method is presented in Supplementary Figure 2 in addition to the supplementary results based on the geometric method. The percentage of HB voxels falling outside the participants' task fMRI FOV was minimal, ranging between 0 and 7.38% (*M* = 1.06, *SD* = 2.39) for bilateral HB masks based on image intensity and between 0 and 7.56% (*M* = 1.08, *SD* = 2.25) for bilateral HB masks created via the geometric method.

Following HB delineation, the fMRI data subjected to ROI analyses were smoothed using a 2 mm FWHM, whereas the fMRI data used for analysing the whole FOV were normalised to MNI space and subsequently smoothed with a 6 mm FWHM. The usage of different smoothing kernels is in line with previous research on HB activity (Lawson et al., 2013, 2014).

Additional motion regressors were created using the Artefact Detection Toolbox (ART, http://www.nitrc.org/) with cut-offs reflecting the 97th percentile which are suited for our voxel size (global signal change > 5, translation > .9 mm). For the creation of physiological regressors relating to cardiac and respiratory effects, TAPAS R2019b as implemented within the MATLAB PhysIO Toolbox (Kasper et al., 2017) was utilized. The first level analyses additionally incorporated the six motion regressors and the changes in translation and rotation between subsequent volumes. The first level model included each MID component (Cue Loss, Cue Neutral, Blank, Reminder Loss, Reminder Neutral, Arrow, Outcome Loss Avoidance, Outcome Loss, Outcome Neutral, Fixation) and all events were modelled with a boxcar function.

### 2.5 Statistical analyses

The whole FOV fMRI analyses were carried out per experimental MID phase (Cue, Anticipation, Outcome) and based on the activation differences (e.g., Anticipation phase: Loss–Neutral) calculated at the first level. These contrasts were then included at the group level to perform one-sample *t*-tests, which were thresholded at *p*_uncorrected_ < .001. Statistical differences were defined as *q* < .05 at the cluster level following False Discovery Rate (FDR) correction.

For the HB specific analyses, percent signal change was extracted for left HB, right HB and the combined bilateral HB masks using Marsbar (Brett et al., 2002) and analysed with SPSS v15. Separate repeated-measures ANOVAS (rmANOVA) on the individual MID phases were run per ROI with Greenhouse-Geisser correction applied when applicable and corresponding significant post-hoc *t*-tests were Bonferroni-corrected (*p*_c_).

### 2.6 Task-based functional connectivity of the habenula

To enable the assessment of functional connectivity between the left and right HB and the whole FOV at the group level, habenula seed ROIs, separate for left and right, were created by manually selecting each individual habenular voxel on the study-specific T1w template (in MNI space) using the software MRIcron (Rorden & Brett, 2000). Functional connectivity between the HB seed regions and the whole FOV was assessed using the generalized task-dependent psychophysiological interaction toolbox (gPPI, http://www.nitrc.org/projects/gppi), which calculates functional connectivity based on the deconvolved first eigenvariate of the seed time series. Functional connectivity was assessed while correcting for physiological variables and motion as described for the activation analyses and using the same statistical thresholds and one-sample *t*-tests on the contrasts between conditions.

## 3 Results

### 3.1 Behavioural

Behaviourally, participants responded correctly and on time to 47% of loss trials and 44% of neutral trials. Response times for correct trials were significantly faster for loss (*M* = 321.06, *SD* = 37.85 msec) than neutral (*M* = 361.11, *SD* = 40.55 msec) trials [*t* (17) = 5.96, *p* < .001]. Concurrently, the performance-based response window duration was significantly shorter during loss (*M* = 360.97, *SD* = 46.08 msec) trials than neutral (*M* = 398.70, *SD* = 45.63 msec) trials [*t* (17) = 5.40, *p* < .001]. For 17 participants, data were available to delineate the most common type of wrong responses, which were correct responses occurring outside the response window for loss (*M* = 91.99%, *SD* = 6.83) as well for neutral trials (*M* = 95.02%, *SD* = 6.16).

### 3.2 fMRI whole FOV

**Fig. 3:**
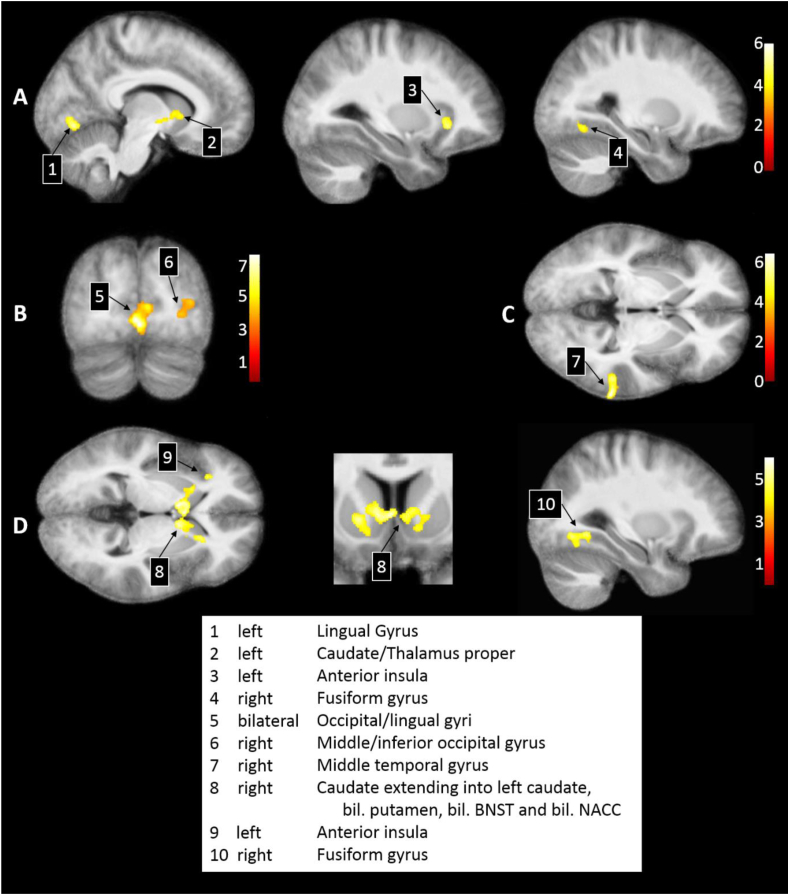
Significant cluster and peak level differences during the MID phases. A) Significantly enhanced activation during Loss compared to Neutral Cues. B) Significantly higher activation in Neutral compared to Loss avoidance Outcomes. C) Significantly enhanced activation during monetary Loss compared to Neutral Outcomes. D) Significantly enhanced activation during Neutral compared to monetary Loss outcomes. Numerals reflect the cluster numbers and correspond to the labels at the bottom of the figure, more information can be found in Table 1 per cluster number. The colour legends indicate the significance level (*T* statistics). All activations are projected onto the 7 T study-specific MNI template.

Cluster level statistics and peak locations are provided in Table 1. Comparing the loss and neutral cues revealed significantly higher activation to loss cues in four clusters, two occipital, one in the left anterior insula, and one in the left caudate extending into the thalamus proper (see Fig. 3a). No significant clusters were found for the reverse contrast.

**Table 1:** Clusters expressing significantly different activations across Loss and Neutral conditions per MID phase.

Contrast	Cluster number	Hemisphere	Location	*q*_FDR_	MNI coordinates (x, y, z)	*z*	k_E_
Cue: Loss - Neutral	1	left	Lingual gyrus	.005	−11, −75, −7	4.33	1045
	2	left	Caudate extending into thalamus proper	.025	−12 6 0	3.95	598
	3	left	Anterior insula	.031	−28 24 -4	3.92	512
	4	right	Fusiform gyrus	.025	32 -66 -6	3.75	561
Outcome: Neutral – Loss Avoidance	5	bilateral	Occipital/lingual gyri	<.001	1, −80, −4	4.96	3999
	6	right	Middle/inferior occipital gyrus	.029	36 -82 10	3.85	678
Outcome: Monetary Loss - Neutral	7	right	Middle temporal gyrus	.036	68 -37 -2	4.53	647
Outcome: Neutral – Monetary Loss	8	right	Caudate extending into left caudate, bil. putamen, bil. BNST and bil. NACC	<.001	20 22 -4	4.28	3147
	9	left	Anterior insula	.023	−29 30 2	3.87	574
	10	right	Fusiform gyrus	.023	30 -66 -7	3.81	575

*Note.* Shown are the significant clusters, their lateralization, their location according to SPM's Neuromorphometrics atlas, the corresponding significance following False Discovery Rate correction (*q*_FDR_), the coordinates of the peak voxel within each cluster according to MNI space, the corresponding *z* statistic, and cluster extent (k_E_).

Next, the activation differences between loss and neutral anticipation phases was assessed. While on the cluster level no significant differences remained following FDR correction, the right inferior frontal gyrus was marginally more active during anticipation of loss than neutral trials (*q*_FDR_ = .056, *z* = 4.35, k_E_ = 761, MNI: 43, 40, 1). No significant clusters or peaks were detected for the reverse contrast.

To compare the neural activation patterns during the MID outcome phase, first, loss avoidance outcomes were compared to neutral outcomes. Whereas the reverse contrast did not reveal statistically significant clusters or peaks, enhanced activation for neutral over loss avoidance was found for two occipital clusters, one containing the bilateral lingual gyrus, the other the right middle and inferior occipital gyrus, shown in Fig. 3b. Next, monetary loss outcomes were compared to neutral outcomes. Significantly higher activation to monetary loss than neutral outcomes was found for one cluster in the right middle temporal gyrus, see Fig. 3c. In terms of increased neural activation to neutral than to monetary loss outcomes, three significant clusters were identified. We observed a decrease in activity to monetary loss in the left anterior insula, the right fusiform gyrus and in the right caudate. The caudate cluster originated in the right hemisphere, but contains bilateral caudate, bilateral putamen, bilateral NACC as well as subpeaks in the bilateral bed nucleus of the stria terminalis (BNST), see Fig. 3d.

No significant differences emerged on peak or cluster level when comparing loss avoidance to monetary loss outcomes.

### 3.3 fMRI habenula

The individual rmANOVAS on percent signal change during cue [right HB: *F* (1,17) = .51, *p* = .49, left HB: *F* (1,17) = .01, *p* = .94, bilateral HB: *F* (1,17) = .19, *p* = .67] and anticipatory phases [right HB: *F* (1,17) = 2.80, *p* = .11, left HB: *F* (1,17) = .33, *p* = .57, bilateral HB: *F* (1,17) = 1.25, *p* = .28] did not reveal differences between neutral and loss trials.

**Fig. 4:**
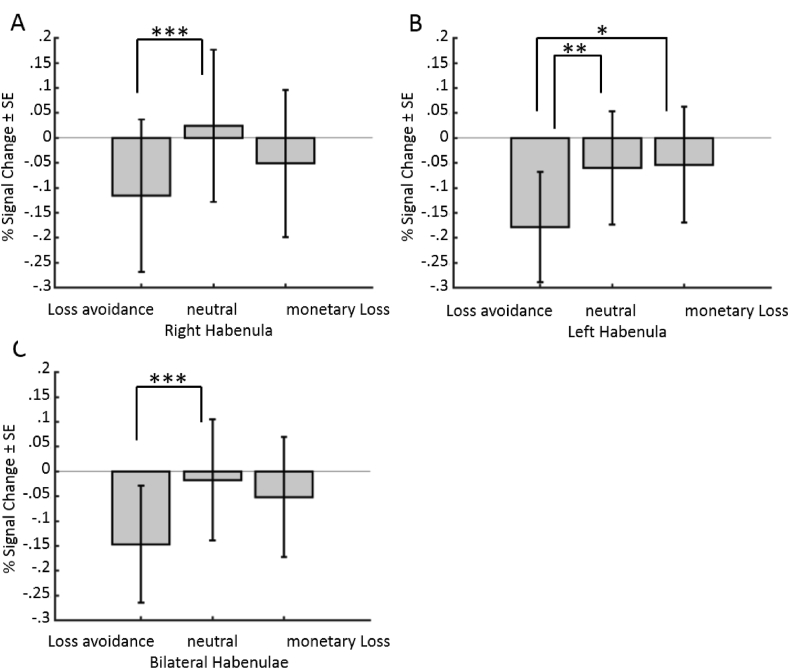
Shown are means and standard errors (SE) of the percentage signal change during the MID outcome phase in the habenulae drawn based on image contrast. A) right habenula, B) left habenula, C) bilateral habenulae. All bar graphs are separated into Loss avoidance, Neutral, and monetary Loss outcomes. Significance levels are indicated by asterisks, where ∗ equals *p* < .05, ∗∗ equals *p* < .01 and ∗∗∗ refers to *p* < .001.

The rmANOVA on percent signal change during the outcome phase, divided into neutral, loss avoidance and monetary loss outcomes, revealed a main effect of outcome type (see Fig. 4) for the right HB [*F* (2,34) = 7.68, *p*_c_ < .01), the left HB (*F* (2,34) = 4.95, *p*_c_ < .05] and bilateral HB [*F* (2,34) = 7.17, *p*_c_ < .01] following Bonferroni-correction. Post-hoc comparisons indicated lower activity during loss avoidance outcomes (right HB: *M* = −.12, *SD* = .65; left HB: *M* = −.18, *SD* = .47, bilateral HB: *M* = −.15, *SD* = .50) as compared to neutral (right HB: *M* = .02, *SD* = .65; left HB: *M* = −.06, *SD* = .48, bilateral HB: *M* = −.02, *SD* = .52) outcomes for the right (*t* (17) = 4.85, *p*_c_ < .001), left [*t* (17) = 3.48, *p*_c_ < .01], and bilateral HB [*t* (17) = 4.56, *p*_c_ < .001].

Enhanced signal change was also observed during monetary loss (right HB: *M* = −.05, *SD* = .63; left HB: *M* = −.05, *SD* = .49, bilateral HB: *M* = −.05, *SD* = .51) as compared to loss avoidance outcomes for the left HB [*t* (17) = 2.86, *p*_c_ < .05], while not significant for the right HB [*t* (17) = 1.53, *p* = .144]. The difference between loss avoidance and monetary loss in the bilateral HB did not remain significant following Bonferroni correction [*t* (17) = 2.65, *p*_c_ = .05]. In other words, monetary loss outcomes were associated with significantly increased left habenula activity compared to avoiding loss or a potentially rewarding outcome.

The comparisons between neutral outcomes and monetary loss was not significant for right [*t* (17) = 2.16, *p*_c_ = .14], left [*t* (17) = .11, *p* = .912] or bilateral HB [*t* (17) = .84, *p* = .41].

### 3.4 Task-dependent functional connectivity with the habenula

Comparing functional connectivity as indexed by gPPI during the cue phase across loss and neutral conditions did not reveal significant coupling differences between the left or right HB and other regions at *q*_FDR_ < .05.

**Fig. 5:**
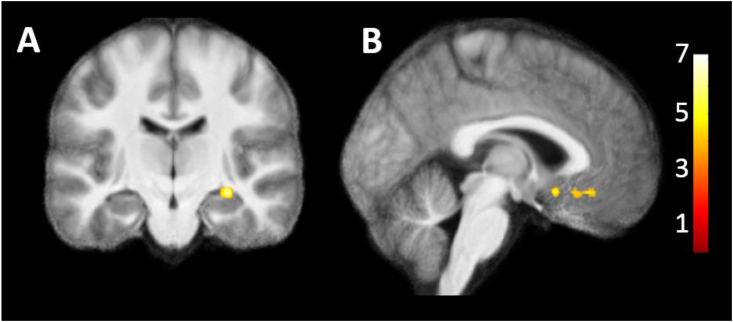
Shown are the regions being characterised by significant positive gPPI slopes with the right habenula when comparing Loss to Neutral Anticipation phases. A) right hippocampus, B) subcallosal cingulate.

When comparing loss anticipation to neutral trial anticipation, a significant positive slope represented the relationship between the right HB and the right hippocampus (*q*_FDR_ < .05, *z* = 4.87, k_E_ = 510, MNI: 31–20 -12), see Fig. 5a. Similarly, a significant positive slope was found for the relationship between the right HB and the subcallosal cingulate (*q*_FDR_ < .05, *z* = 3.94, k_E_ = 514, MNI: −2 13–12), see Fig. 5b.

When contrasting the functional connectivity across different outcome types, no significant differences in functional connectivity between the left and right HB seeds and other brain areas were found.

## 4 Discussion

In this first ultra-high field neuroimaging study on human HB function during loss avoidance, we show activity as expected of the HB given its upstream role in modulation of midbrain dopaminergic function (Matsumoto & Hikosaka, 2007). We showed decreased HB activity, in both hemispheres, to loss avoidance outcomes (which are effectively acting as reward, relative to neutral and monetary loss). Behaviourally, response times were faster during loss avoidance than neutral trials indicating enhanced motivation and potentially salience of these trials. Greater left HB activity was also observed during monetary loss versus loss avoidance outcomes. During anticipation of loss relative to neutral outcomes, we found enhanced right HB functional connectivity with the subcallosal cingulate (SCA) and hippocampus.

In the whole FOV approach, even at ultra-high field (7 T), HB-specific activation was not identifiable due to cluster size thresholding and multiple comparison corrections and larger smoothing kernels, further highlighting the need for an ROI approach to investigate HB functionality (Lawson et al., 2013). Single-cell LHB recordings have shown greater firing to the most negative outcome among alternatives and with inhibition to reward, especially at low predictability (Matsumoto & Hikosaka, 2009). Similarly, human 3 T fMRI studies showed enhanced HB activity to both aversive shock and when comparing loss relative to reward outcomes (Furman & Gotlib, 2016; Lawson et al., 2014). Loss avoidance outcomes here behave similarly to reward outcomes: a deactivation of HB would be presumably associated with greater midbrain dopamine release. The monetary loss outcomes also show greater activity than loss avoidance outcomes which presumably would be associated with a cessation of midbrain dopamine activity (Fiorillo et al., 2003).

Our primary whole-FOV findings were in the outcome phase, demonstrating that monetary loss relative to neutral outcomes was associated with deactivation of bilateral caudate, putamen, BNST and NACC, and the left anterior insula. The NACC, caudate and putamen have previously been shown to be differentially responsive to the anticipation of neutral and monetary loss trials with putaminal activity further reported in the MID outcome phase (Knutson et al., 2001). The NACC and anterior insular activity have previously been associated with tracking negative prediction error (Harrison et al., 2016; Voon et al., 2010).

Loss cues in the whole-FOV analysis were associated with activity in regions implicated in loss and value representation with greater predominantly left-sided activity in the anterior insula and caudate. The loss cue, although associated with the opportunity to avoid losing, predicted an increased chance on losing than the neutral cue.

In contrast to previous meta-analyses which have shown similar activations during the anticipatory phase of reward and loss trials in the MID task (Dugre et al., 2018; Oldham et al., 2018; Wilson et al., 2018), we did not observe any differential activity in the anticipation phase in the whole-FOV analyses. However, we found differences in functional connectivity with greater connectivity between the right HB and hippocampal and subcallosal cingulate. The hippocampal involvement likely reflects underlying memory-related processing (Savage et al., 2004) and has been reported during the anticipatory phase in the MID task (Patel et al., 2013). Hippocampal activity has been previously shown to linearly scale with loss magnitudes (Hahn et al., 2010). Rodent lesion studies suggest that the hippocampal complex is especially relevant at learning the initial matching between cues and outcomes as well as during memory processing relating to non-specific reward expectancy (Savage et al., 2004). The role of the hippocampus may relate to information transfer between the anticipatory cue and associated potential outcomes.

The SCA is a projection target for midbrain dopaminergic neurons modulated by HB activity (Matsumoto & Hikosaka, 2007; McInerney et al., 2017). Resting-state 3 T functional connectivity of the human HB has previously identified enhanced functional coupling between the HB and the SCA (Erpelding et al., 2014). Similarly, a probabilistic Pavlovian learning paradigm with monetary rewards, losses and electric shocks as punishment showed a non-significant increase in functional coupling between the right HB and Brodmann Area 25, with increasing motivational value of the punishment-related conditioned stimulus (Lawson et al., 2014).

Our findings might be particularly relevant in the context of major depression. Depression is associated with abnormal subcallosal cingulate activity and connectivity patterns (Greicius et al., 2007; Mayberg et al., 2005; Roiser et al., 2009; Tozzi et al., 2017) and positive effects on depressive symptoms were reported with deep brain stimulation (DBS) targeting the SCA (Holtzheimer et al., 2012). A similar remission of depressive symptoms has been seen in a case study following DBS to the lateral HB in an MDD patient (Sartorius et al., 2010) and in a patient with bipolar disorder with refractory depression (Zhang et al., 2019).

The current study is not without limitations. In light of the intended functional connectivity and FOV brain analyses in addition to the HB ROI analyses, we attempted to include core MID task-related structures such as the hippocampus and subcallosal cingulate in the assessed FOV. This, meant that we could not centre the FOV over the HB, which enabled one participant to move their HB out of the FOV during the scan and corresponding data was subsequently discarded from all analyses. We were also unable to distinguish between lateral and medial HB. Further, HB ROIs were not independently confirmed by other raters. We address this issue by presenting results of ROIs created two ways: based on 7 T image contrast and using the geometric method (Lawson et al., 2013). Finally, while we controlled for known confounds, such as cardiac rhythm and respiratory rate (Hutton et al., 2011), recent evidence also hints towards a possible effect of circadian rhythm (Kaiser et al., 2019). Given the divergent findings in the literature regarding lateralization of HB function, we chose to analyse left, right and bilateral HB separately without directly assessing lateralization. Of note, the majority of trials leading to negative feedback in the loss condition were correct responses occurring outside the response window. While staircase procedures, hence adjusting the allowed response window, are commonly utilized in MID tasks [e.g., (Mei et al., 2018; Miller et al., 2014; Nestor et al., 2017; Zhang et al., 2017)], incorrect button presses occurred at a low rate. As such we suggest that investigations aiming to specifically delineate the HB response to behavioural errors utilize task designs evoking higher proportions of incorrect button presses.

In summary, we demonstrate that HB activity differentiates between monetary loss avoidance, monetary loss and neutral outcomes for the first time in an ultra-high field (7 T) subcortical task-based fMRI study. Our findings thus converge with proposed HB function in rodent studies and extend previous observations in human imaging studies at 3 T. The HB appears to be a critical structure particularly in depressive disorders and has been implicated as a potential key node in the anti-depressive mechanism of action of ketamine (Yang et al., 2018b). Further studies using task-based fMRI at 7 T to investigate the role of the HB in depression and the effects of ketamine are warranted.

## Funding and disclosure

The authors declare no conflict of interest. This research was supported by the NIHR Cambridge Biomedical Research Centre (BRC-1215-20014). VV is funded by a Medical Research Council Senior Fellowship (MR/P008747/1). CLN is funded by the Medical Research Council (Grant Reference: SUAG/043 G101400). CTR is funded by a Sir Henry Dale Fellowship from the Wellcome Trust and the Royal Society [098436/Z/12/B]. The 7 T MRI scanner was funded by the University of Cambridge, the Medical Research Council (MR/M008983/1), and the NIHR Cambridge Biomedical Research Centre. The views expressed are those of the author(s) and not necessarily those of the NIHR or the Department of Health and Social Care.

No part of the study procedures and analyses was pre-registered prior to the research being conducted. We report how we determined our sample size, all data exclusions, all inclusion/exclusion criteria, whether inclusion/exclusion criteria were established prior to data analyses, all manipulations, and all measures in the study. Study data, digital study materials, and analysis code are available via https://doi.org/10.17863/CAM.66358.

## Author contributions

KW supported all aspects of the project. SGK and CLN supported the data analyses. CR and CTR supported the data acquisition. VV acquired the funding and supported study design and analyses. All authors supported the drafting of the manuscript and agree to this publication.

## Open practices

The study in this article earned Open Data and Open Materials badges for transparent practices. Data and Materials for this study can be found at https://doi.org/10.17863/CAM.66358.
