# Supplementary material for: Avoiding monetary loss: A human habenula functional MRI ultra-high field study
Source: Cortex. 2021 Sep;142:62–73. doi: 10.1016/j.cortex.2021.05.013 (PMC8422162; doi:10.1016/j.cortex.2021.05.013)
Supplement: Multimedia component 1 [file mmc1.docx]

***Supplementary Material***

**Methods**

**fMRI HB: using geometric masks**

To further support our results, we additionally created the habenulae (HB) masks based on the geometric method^3^ in MRIcron^31^. In the geometric method, the habenula outline is created by defining three points, A, B and C (see Supplementary Figure 1). Point A indicates the junction between the posterior commissure/habenular commissure and the medial edge of the habenula. Point B reflects the most dorsal point of the HB where the HB intersects with the medial dorsal thalamus. Point C is reflecting the lateral junction between the midbrain and the pons. Following creation of the HB outline, each individual voxel within that outline was manually selected to create separate ROI masks for right, left and bilateral HB. The subsequent preprocessing/analyses steps were identical to those reported in the main document for HB masks created based on image intensity. See Supplementary Figure 2 for HB masks created via visual inspection of image intensity and the geometric method.

**Results**

**fMRI HB: using geometric masks**

The individual rmANOVAS on percent signal change during Cue (right HB: *F*(1,17) = 70, *p* = .42, left HB: *F*(1,17) = .02, *p* = .90, bil. HB: *F*(1,17) = .20, *p* = .66) and Reminder phases (right HB: *F*(1,17) = 3.21, *p* = .09, left HB: *F*(1,17) = .33, *p* = .58, bil. HB: *F*(1,17) = 1.40, *p* = .25) were not significantly different between neutral and loss trials.

The rmANOVA on percent signal change during the Outcome phase, divided into neutral outcomes, loss avoidance and monetary loss outcomes (see Supplementary Figure 3), revealed a main effect of outcome type for the right HB (*F*(2,34) = 9.21, *p* < .001), the left HB (*F*(2,34) = 3.79, *p* < .05), and bil. HB (*F*(2,34) = 6.92, *p* < .01). Post-hoc comparisons indicated a significant difference between loss avoidance (right HB: µ = -.16, *SD* = .68; left HB: µ = -.16, *SD* = .45, bil. HB: µ = -.15, *SD* = .50) and neutral (right HB: µ = -.01, *SD* = .67; left HB: µ = -.05, *SD* = .45, bil. HB: µ = -.02, *SD* = .51) outcomes for the right (*t*(17) = 4.98, *p*_c_ < .001), left (*t*(17) = 3.06, *p*_c_ < .05), and bil. HB (*t*(17) = 4.43, *p*_c_ < .01).

Statistically significant differences between loss avoidance and monetary loss (right HB: µ = -.09, *SD* = .65; left HB: µ = -.05, *SD* = .47, bil. HB: µ = -.07, *SD* = .52) conditions were absent for right (*t*(17) = 1.60, *p* = .13) and did not remain significant for left (*t*(17) = 2.43, *p*_c_ = .08) and bil. HB (*t*(17) = 2.61, *p*_c_ = .10) following Bonferroni correction.

The comparisons between neutral outcomes and monetary loss was not significant for left (*t*(17) = .003, *p* > .99) and bil. HB (*t*(17) = 1.14, *p* = .27), and did not remain significant following Bonferroni correction for right HB (*t*(17) = 2.60, *p*_c_ = .06).


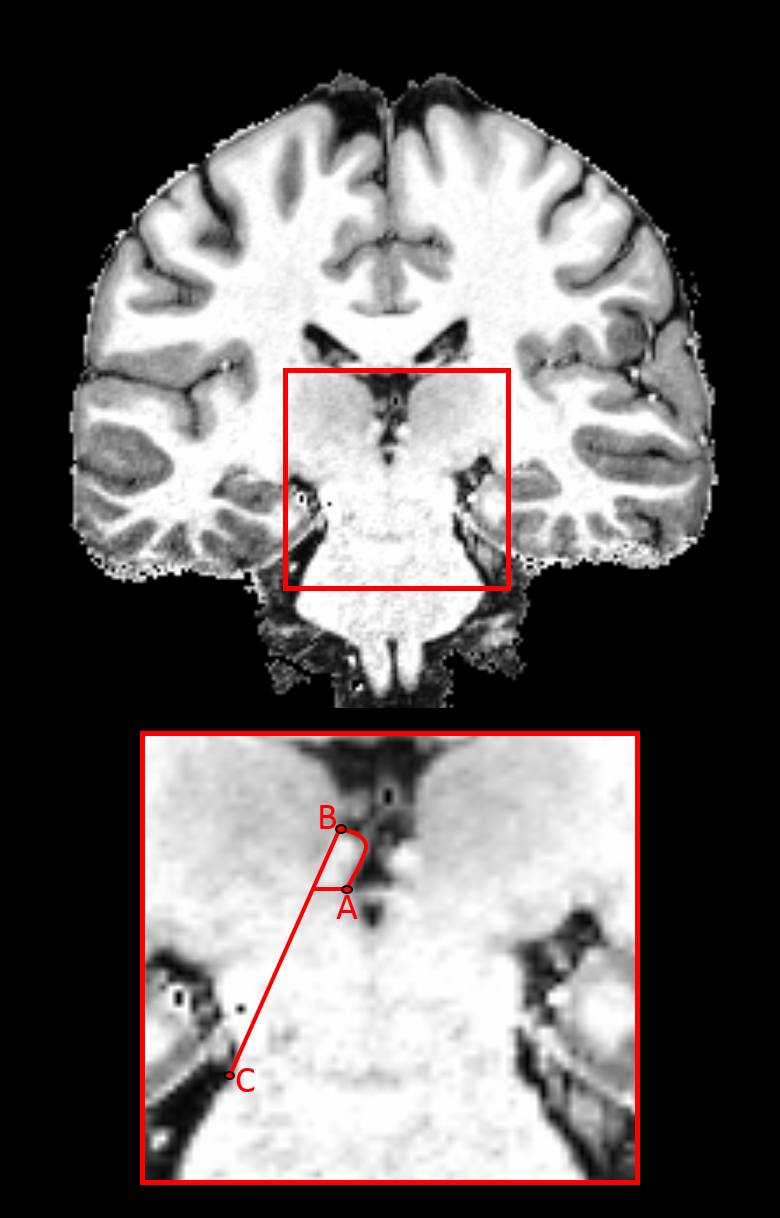


*Supplementary Figure 1.* Shown are the points defining the outline of the human habenula based on the geometric method^3^. Point A = junction between posterior commissure/habenular commissure and medial edge of the habenula, Point B = most dorsal point of the habenula that intersects with the medial dorsal thalamus, Point C = lateral junction between midbrain and pons.


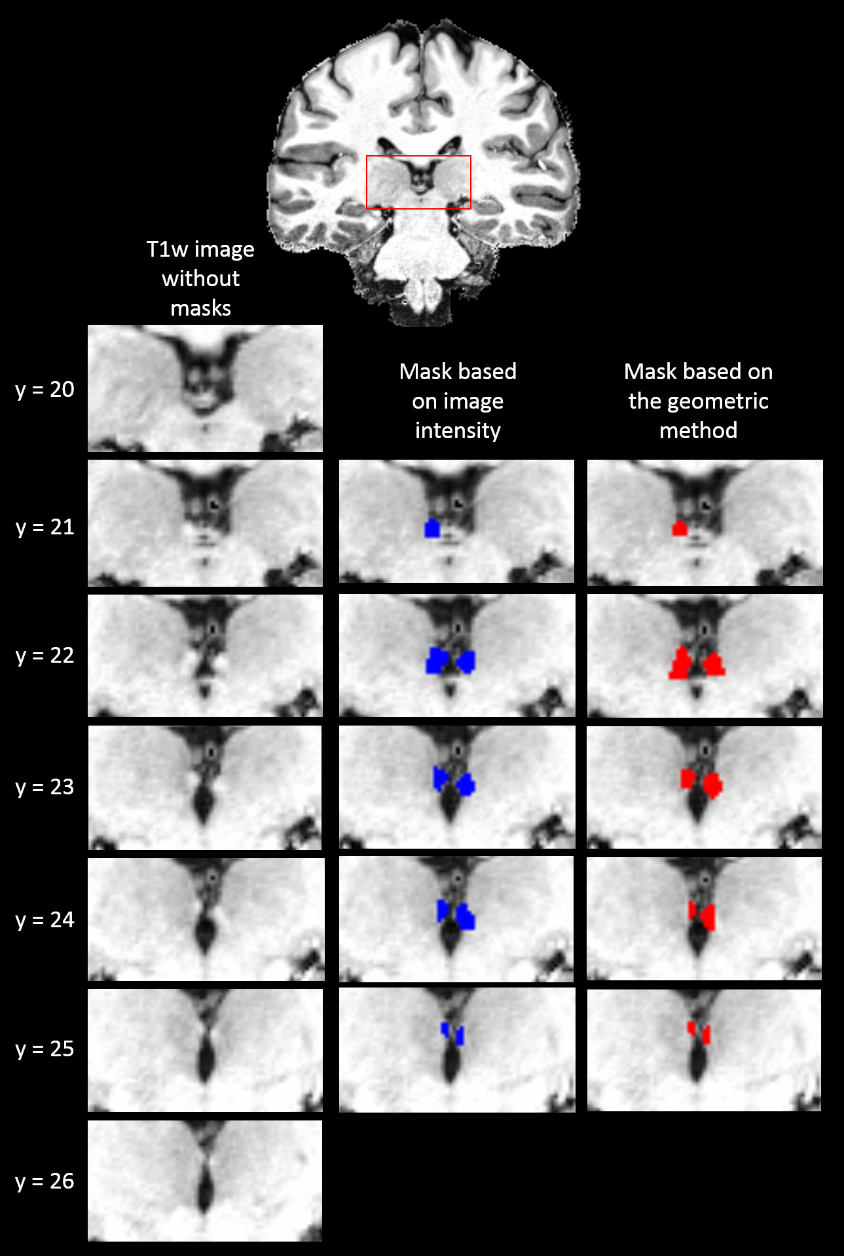


*Supplementary Figure 2.* Examples of habenula (HB) masks created in MNI space for a representative participant. The first column shows the image intensity obtained for the habenulae at 7T with the MP2Rage (without overlaid HB masks). The second column shows the masks created when relying on image intensity to define voxels belonging to the HB. The third column shows the masks created based on the geometric method^3^. Note that differences are minimal and mainly present in the ventral and lateral edges of the HB in more posterior slices (e.g. y = 21, 22 mm) where 3T image intensity is usually insufficient to delineate the HB outline based on image intensity alone.

*
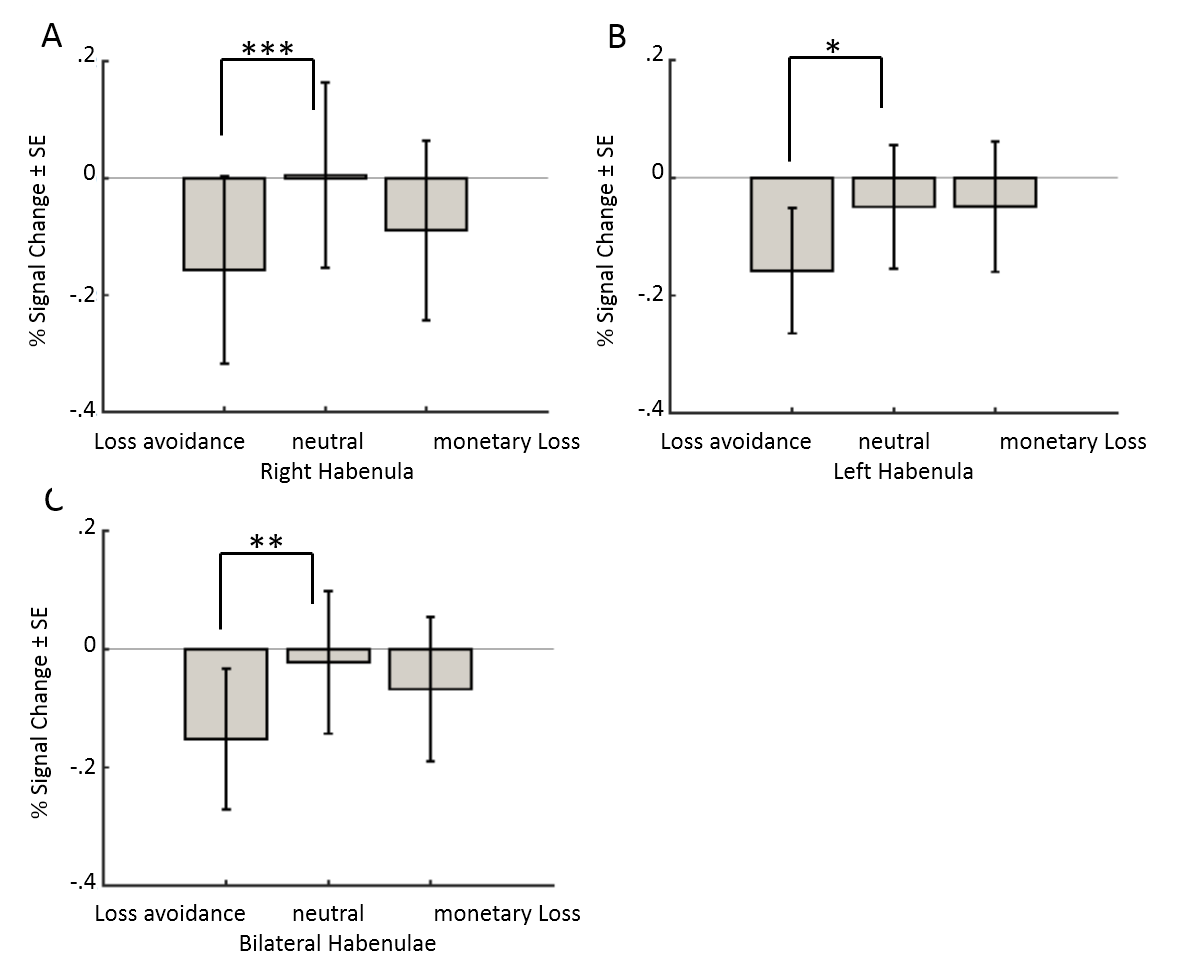
*

*Supplementary Figure 3.* Shown are means and standard errors (SE) of the percentage signal change during the MID outcome phase in the habenulae ROIs created via the geometric method. A) right habenula, B) left habenula, C) bilateral habenulae. All bar graphs are separated into Loss avoidance, Neutral, and monetary Loss outcomes. Significance levels are indicated by asterisks, where * equals *p* < .05, ** refers to *p* < .01, and *** indicates *p* < .001.
